# Relationships between Body Composition Parameters and Phase Angle as Related to Lifestyle among Young People

**DOI:** 10.3390/jcm11010080

**Published:** 2021-12-24

**Authors:** Aleksandra Jaremków, Iwona Markiewicz-Górka, Wojciech Hajdusianek, Paweł Gać

**Affiliations:** 1 Department of Hygiene, Wroclaw Medical University, 7 Mikulicza-Radeckiego St., 50-345 Wroclaw, Poland; iwona.markiewicz-gorka@umw.edu.pl (I.M.-G.); wojtek243@gmail.com (W.H.); pawel.gac@umw.edu.pl (P.G.); 2 Department of Population Health, Division of Environmental Health and Occupational Medicine, Wroclaw Medical University, 7 Mikulicza-Radeckiego St., 50-345 Wroclaw, Poland

**Keywords:** body composition, lifestyle, phase angle, students

## Abstract

The aim of the study was to indicate which body composition parameters and which lifestyle components affect the phase angle (PA) value in young adults. Two-hundred-and-eighty-one students at Wroclaw Medical University participated in the study. A survey on respondent lifestyles was followed by anthropometric measurements, body composition analysis, and basal metabolic rate (BMR) calculation. The differences in the body composition of men and women corresponded to their sexual anatomy and physiology. Sex-dependent differences in lifestyle were also reported. The study revealed a relationship between PA and height, weight, BMR, BMI (body mass index), FFM (fat-free body mass), bone mass, water mass, muscle mass (r~0.4–0.7), ECW/ICW (extracellular water/intracellular water) and fat mass (r~−(0.4–0.6)) for the entire studied group. The relationship between PA values and lifestyle components concerned consumption of energy drinks, cola, alcohol, water, vegetables and fruits, meat, and also intervals between meals, time for physical exercises, and screen time (r~±0.2). The research shows that the PA value increases with an increase in positive body composition parameters. Following the principles of proper nutrition and physical activity increases PA values in most cases.

## 1. Introduction

The quantitative assessment of body composition (such as adipose tissue mass, muscle mass, body water bass, fat-free mass, etc.) can be done by an analyser based on the bioelectrical impedance analysis (BIA) technique. BIA is an uncomplicated and non-invasive method based on measuring the resistance (R) and reactance (capacitive resistance Xc) of soft tissues and consequently the tissue’s electrical resistance. An electrical current with low amperage and high frequency is passed through the human body by electrodes placed on the weighing scale platform connected to the feet and in handles held in both hands [1]. Since the current flows easier through electrolyte solutions than cells, the resistance measured by adipose tissue or extracellular water causes a voltage drop. On the other hand, cell membranes, which are composed of two lipid layers, do not conduct electricity but rather act as a capacitor accumulating electric charges on each side. Therefore, it is possible to measure reactance responsible for the phase shift of the applied electric current expressed as phase angle (PA) [2]:

PA = arc tangens Xc/R(1)

A high PA value indicates the high electric potential of a cell membrane, and, therefore, it means appropriate nourishment of body cells, which ensure their proper functioning through their building resources and energy reserves. The PA value should be within 5–7 degrees [3] (although reference values may vary slightly depending on the studied population [4]). A decrease in PA indicates a decline in membrane integrity followed by changes in their permeability or amount of extra- and intracellular fluid. Consequently, membranes no longer function properly, which may lead to cellular apoptosis. The efficiency of energy and proteolysis processes, which determine the human health condition, is falling. This may indicate an ongoing chronic or neoplastic disease, malnutrition, high physical exhaustion, or it may happen during a course of sepsis [3].

Taking into consideration the tissues’ electrical properties, PA values will be determined by many parameters of body composition. Although this subject has been analysed in the available literature, the information obtained is not clear. Previous studies show that the level of PA is influenced by age, sex, and BMI (body mass index). A study by Gonzales et al. [5] showed that PA is significantly determined by fat-free mass (FFM), height, age, race, and ECW:ICW (extracellular water/intracellular water) ratio. Furthermore, Siddiqui et al. [6] described the relationship between the PA and body weight, muscle mass, visceral fat, and height. According to Barbosa-Silva et al. [4] the percentage of fat has a significant influence on PA value. PA can also be a good indicator of muscle performance in both young [7] and elderly [8] people. When subjected to physical exercise, older people present an increase in PA, and after their cessation, PA declines [9]. Generally, there is a higher PA in people who regularly practice sports or athletes than in physically inactive people [10]. PA is also correlated with mortality: higher values tend to prognose better outcomes for severely ill patients [11]. However, there is no information on other factors that influence PA—for example lifestyle components. Only the influence of the Mediterranean diet on PA has been investigated already, obtaining a positive correlation [12].

The aim of the study was to show which parameters of body composition and which lifestyle components correlate with PA values in the group of young people. The obtained result may contribute to a decrease of negative health behavioural patterns among them and therefore improve their quality of life.

## 2. Materials and Methods

### 2.1. Study Design

The study group was composed of 281 Wroclaw Medical University students from the faculty of medicine and faculty of dentistry. The study consisted of filling in the questionnaire, measurements of body composition, basal metabolism rate, and anthropometrics.

### 2.2. Questionnaire Survey

Students who agreed to participate in the study filled in a paper-based questionnaire about their current lifestyle. The questions concerned mainly eating habits (How often do you drink energy drinks/coffee/Cola/various types of alcoholic beverages? How much water do you drink a day? What is the time interval between meals? How many meals do you eat per day? How often do you snack between meals? How often do you eat fruit, vegetables/grain products/dairy products/meat/fish/fast food/sweets/? How often do you take supplements?). There were also questions about sleep quality (How many hours do you sleep at night? How often do you take a nap during the day and how long does it take?), regularity of sport exercises (How often and how long do you exercise for?), perceived stress (How often do you feel stressed?) and study and screen time (How much time do you spend studying/watching TV/using a smartphone/computer each day?).

The main part of the survey on the consumption of various food products was assessed for reliability (validation of the questionnaire). The Cronbach’s alpha coefficient was: 0.51 which may be due to the construction heterogeneity of the questions used.

### 2.3. Body Composition Analysis

The body composition analyser TANITA MC-780MA was used in the study. By using bioelectric impedance technology (BIA) it was possible to measure the components of the student’s body and calculate their basal metabolism rate (BMR) and PA value.

In accordance with the recommendations of the manufacturer of the device, the students were measured barefoot in light-weight clothes, without jewellery, and under the following conditions:

- at least 3 h after getting out of bed,
- at least 3 h after a meal,
- at least 12 h after intense physical exercise,
- at least 12 h after drinking alcohol,
- after urinating,
- in women—contraindication: pregnancy and menstruation.

Pacemakers and implants were also excluded from the study due to the possible bias in the measurement (a large impact on the body composition).

### 2.4. Anthropometric Measurements

The height of the participants was measured by using a stadiometer, whereas the circumference of the waist and hips was measured by using a centimetre tape to then calculate the Waist-Hip ratio (WHR). All measurements were made twice to calculate a mean. The types of obesity classification were based on the World Health Organisation standard for WHR (gynoid obesity: WHR < 0.85 (female), WHR < 0.9 (male); android obesity: WHR ≥ 0.85 (female), WHR ≥ (male) [13].

### 2.5. Statistical Analysis

All statistical computations were performed by using the Statistica 13.0 program. The *t*-student test was used to determine, based on sex, differences in the measured anthropometric parameters and body composition. It is important to mention that some of the tests were done after the logarithmic transformation of variables. Furthermore, if a normal distribution of variables was absent, nonparametric tests were used. Components of the lifestyle (quantitative variables) were compared for women and men by using nonparametric tests (U Mann-Whitney test). To determine the relationship between:

(1) anthropometric parameters of body,
(2) components of lifestyle,
(3) PA.

Either Pearson or Spearman correlations were performed (depending on the fulfilment of assumptions for parametric tests). In addition, logistic regression was used to assess the influence of independent parameters on the nutritional status of the organism, which was measured by the PA: 5–7(8). A *p*-value of less than 0.05 (*p* < 0.05) was the limit of statistical significance.

## 3. Results

### 3.1. Characteristics of the Studied Population

The majority of the respondents were second-year students of the faculty of medicine and were about 21 years old. Fifty-eight per cent of the respondents were women while 42% were men. Participants came from towns of various sizes, with a slight predominance of large cities. Those students lived mainly in either rented or owned apartments and single rooms in shared (with other people) apartments. The surroundings of the place of residence tended to be peaceful for more than half of the respondents. The full results on this issue are presented in Table 1.

### 3.2. Sex Differences in Anthropometric and Body Composition

Significant differences in anthropometric and body composition for both sexes are presented in Table 2. Men were characterized by significantly higher height, body weight, BMR value, FFM, water, muscle, and bone mass in their bodies. PA and BMI were higher as well. Unlike women, more men were overweight/obese (24 participants: 8.54%; gynoid obesity—22 people: 7.83%). Only one male student was underweight and the rest (93 people: 33.10%) had normal BMI. In women, BMI was distributed as follows: one person underweight (0.36%); 130 people had normal BMI values (46.26%); 10 people were overweight or obese (gynoid obesity 10 people 3.56%). Unlike men, women had higher body fat and a higher ECW to ICW ratio.

### 3.3. Sex Differences in the Lifestyle Components

Table 3 describes significant differences in the lifestyle of respondents by sex. Men drank energy drinks, cola, beer, spirits-based alcohols, water, and consumed the meat and fast foods significantly more often than women. They also kept greater intervals between meals and spent more time on both physical activity/exercise and using the computer for entertainment purposes. On the other hand, women drank wine and alcoholic drinks significantly more often and ate more fruit and vegetables than men. They also spent a long period in front of their smartphones.

In the case of the remaining survey questions, no significant differences by sex were noted.

### 3.4. Relationship between PA Value and Anthropometrics and Body Composition Parameters

When the whole group was analysed together, positive correlations were found between PA and body weight, water, and muscle content in the body (negative correlation: PA value and a fat mass and fat percentage). Relationships between the values of the PA and BMR, FFM, TBW (total body water), muscle mass, BMI, ECW/ICW, and bone mass were also revealed (men, women, and the whole group). Only height was not significantly correlated with PA in the men group though (Table 4).

### 3.5. Relationship between PA Value and Lifestyle Components

The PA value was positively correlated with cola, beer, spirits-based alcohols, water consumption, meal interval, meat consumption, and frequency of exercise in the whole study group (negative correlation: between PA and consuming alcoholic drinks, fruit and vegetable consumption, and time spent on watching TV, using smartphones). There was also a correlation between PA and energy drink consumption (only in the group of women—no correlation) and time spent on exercise (only in the group of men—no correlation). The full results are presented in Table 5.

### 3.6. Influence of Various Variables on PAValue in the Multivariate Model

The probability of obtaining the correct PA (5–7/8) increased with an increase in body weight and frequency of meat consumption, as well as a decrease in height and fat content in the body (Table 6).

## 4. Discussion

As expected, considering the anatomy and physiology of sexual development, the body composition of men and women differs significantly. Therefore, it is not surprising that the male group had greater values in height, WHR, BMI, weight, BMR, PA, FFM, water mass, muscle mass, and bone mass than in women. In turn, women have higher fat content than men. Those results are natural and result for example from differences in the endocrine system because the amount and distribution of adipose tissue closely relate to oestrogens (such as estrone produced by adipocytes). Women have more and larger fat cells in the subcutaneous tissue, which are mainly located in the gluteal region. On the other hand, although having a greater fat-free mass, men are characterized by a greater fraction of visceral (instead of subcutaneous) adipose cells and have higher WHR [14]. Men tend to be taller and heavier than women, have greater BMI, and consequently a greater number of cells that increase their PA in comparison to women [4]. There is also a difference in water content (typically: 60–63%—M and 52–55%—W) [15]. Since muscles are composed of 75% water, their mass is also greater. The increase in muscle mass improves their capability of work, which relates to an increase in the general metabolism [16]. According to other research [5,17] in our study women present a greater ECW/ICW ratio. This is an integrity of the cell membrane indicator that determines the proper functioning of the cell, its hydration and may be used to assess muscle strength. The ratio is strictly connected to PA: the greater its value, the lower the ECW/ICW ratio [18]. This is confirmed by the results of our study (negative correlation: r = −0.594; r = −0.475; r = −0.254, for the whole study group, for the men, and for the women, respectively). Due to the higher PA value in the men, their ECW/ICW ratio was lower than in women.

Our research shows that healthy eating behavioural patterns vary significantly by sex. Men drink energy drinks and cola considerably more often, which corresponds to numerous studies [19,20,21]. This may be because advertisements for energy drinks are primarily aimed at young men (mainly athletes [22]; according to our research, men spent much more time exercising than women: 72 min/24 h vs. 49 min/24 h).

The frequency of alcohol-based beverage consumption varies as well. In men, beer and strong drinks prevail whereas in women it is wine and mixed drinks, which corresponds to numerous research [23,24]. Following the recommendations, men drank more water (1.7 L/24 h) than women (1.5 L/24 h), although according to the European Food Safety Authority this amount was still not enough (recommendations are: 2.5 L/24 h—M and 2.0 L/24 h—W) [25]. Those differences may be caused by the fact that respondents took into consideration only bottled water, not including the intake of other fluids. Male students also had greater intervals between meals and consumed meat and fast foods more frequently. On the other hand, women were more likely to eat fruit and vegetables. This is confirmed by numerous studies that show that women are more likely to follow the rules of proper nutrition [26,27,28].

The development of technology and technical innovations are becoming more and more important in our everyday life, which was also visible in the results of our study. Students tended to spend approximately 4.5 h daily watching television or in front of the smartphone or computer. Men prefer to watch television and to use the computer whereas women choose to spend time with smartphones. Leisure time patterns may vary depending on sex, which is confirmed in literature [29,30,31].

Due to the fact that PA indicated the overall health condition of the body (cell membrane integrity, cell nutrition status, energetic and proteolysis process efficiency), our aim was to research what influences its value: which parameters of body composition or lifestyle components matter. Therefore, numerous correlation analyses were performed to research a relation between PA and studied factors. It shows that the height was strictly correlated with PA—in the case of women only, we obtained a negative correlation. That is, the greater height they achieve, the lower the measured PA value (Gonzales et al. [5] and Siddiqui et al. [6] obtained similar results). Moreover, a positive correlation was also present with weight, BMI, BMR, FFM, body water content/weight, bone mass, and muscle content/mass. Relationships for body weight and height result from the somatic development of the body—bone growth, muscle growth, more water mass, which together causes an increase in FFM and, consequently, increase PA value. Furthermore, available sources state that men with a similar height as women and simultaneously higher FFM tend to have greater PA value [6] (that is the reason for negative correlation: height—PA in females and positive correlation for the whole study group itself in our study). Similar to body weight, BMI was also positively correlated with PA. According to Bosy-Wesphal [32], with the increase of BMI, the number of cells in the body rises (e.g., muscle cells in athletes) and this is the reason for the PA increase. In addition, a strong correlation of PA with BMR is related to FFM [33] and muscle mass. With the work of muscles, energy is processed, and metabolism increases [16] and then PA as well, which as it turns out is also an indicator of this process [7,8].

Our study shows a negative correlation between PA and fat mass in the body. It is consistent with the studies conducted by Barbosa-Silva et al. [4] and Gonzales et al. [5]. The more fat is present in the body (unfavourable parameter), the greater the risk of civilization diseases (obesity, diabetes, cardiovascular diseases). In addition, with such diseases, the PA values decrease, which supports that correlation.

The obtained results for the relationship between the lifestyle components and PA values showed a weak correlation. Surprisingly, the correlation between PA and drinking cola or energy drinks was positive. Though it is important to mention that such consumption was not very popular among the study group (energy drinks about 1.5/month and cola one time per week), and therefore such small consumption may have a positive impact on PA. The effect of consumption of various types of alcoholic beverages (less than 1 per week) on PA can be similarly explained (only in the case of flavoured alcoholic drinks were the results opposite). This is confirmed by Coehlo et al. [34], who showed a strong correlation between low PA value and frequent alcohol consumption (>80 g/24 h/5 years) in their research.

As recommended by the Food and Nutrition Institute [35], students consumed an appropriate amount of bottled water (1.5 L/24 h) and preserved an appropriate interval between meals. On the other hand, their fruit and vegetable consumption were slightly too low (2 times daily) and too often they ate meat (1/daily). It is important to note that consumption of fruit and vegetables had a negative correlation with PA value and meat had a positive. It is possible that respondents did not take into consideration the vegetables present in their sandwiches and rather regarded only vegetables and fruits consumed in salads or separately. Although surprisingly, daily meat consumption increased PA, it should be noted that responders were neither questioned about the type and source of meat nor about the consumed amount of it. This information could resolve doubts about the validity of the obtained results. Nevertheless, meat as a source of wholesome protein provided the body with energy, which may explain its positive effect on PA [36].

The few studies conducted so far on the influence of nutrition on PA value show that, for example, the Mediterranean diet increases this indicator [12]. However, analysis of the impact of individual products on the PA conducted by Brazilian scientists [37] was not so clear. For instance, frequent fruit consumption (at least three portions daily) and low meat consumption (no more than two weekly) increased the risk of acquiring the PA value (similarly to our study). However, after the exclusion of the confounding factor, correct results were obtained—as expected. In addition, the Healthy Eating Index (HEI) was calculated (on the basis of all present nutritional data), which significantly reduced the chance of getting a low PA.

Playing sports has a positive effect on PA value increases muscle mass and burns fat. This is confirmed by numerous studies [9,10,38] including ours, in which we obtained a positive correlation between frequency and length of exercises and the PA value. On the other hand, the decrease of PA was correlated with an increase of time spent on watching television, using the smartphone or the computer for entertainment purposes (negative correlation). This is one of the first studies in which this relationship is statistically significant. Others [34,39,40] confirm the relationship of these parameters, but it is not significant.

However, in the multivariate model concerning the influence of various co-occurring factors on PA value, only height, body weight, fat percentage, and meat consumption frequency were significant. This shows the importance of these variables along with the simultaneous presence of other factors. The obtained results of PA predictors in the multivariate model (height, body mass/BMI) were confirmed in the literature [5,6]. In turn, the relationship between body fat content and PA has so far been obtained only in univariate analyses. The relationship between meat consumption and PA value was presented for the first time.

The limitation of this study is the reliance on participants’ declarations regarding their lifestyle. Therefore, the control of these variables might be difficult. Moreover, the extrapolation of the obtained results to other groups of people is limited due to the study of only a small group of students from two different faculties. The bioelectrical impedance method used may also be a limitation in our study, especially when measuring obese people. In their case, there are differences in the body geometry and water distribution in the body compared to people with normal body weight. Therefore, the assumed hydration factor could be inappropriate. In our study, obese people constituted a small percentage of the total number of respondents (12.10%) which minimized the negative impact of the indicated measurement error on the obtained results.

## 5. Conclusions

It is well known that sex difference exists in anthropometric and body composition. In this study, we further know that PA value is higher among men than among women. On the contrary, ECW/ICW is higher for women. PA is an important indicator of the proper functioning of the cell in the human body. Therefore, PA is influenced by numerous body composition parameters.

The strength of their relationships with PA for the entire study group ranges from average to very strong (r~0.4–0.7); the strongest correlated: BMR, FFM, TBW, muscle mass. These are strictly interdependent variables, and their positive correlation with PA proves the role of this indicator in muscle work. On the other hand, PA’s relationship to lifestyle components is correlated weakly (r~0.1–0.3). This may be caused by other factors that have not been analysed in this study and the construction heterogeneity of the questions used. Nevertheless, presented results show that compliance with the principles of proper nutrition and appropriate physical activity influence PA values positively.

## Figures and Tables

**Table 1:** Characteristics of the studied population/*n* (%), Mean ± SD/; *n* = 281.

Characteristic	Mean ± SD or *n* (%)
Age [Mean ± SD]	
Total	20.7 ± 1.5
Men	20.7 ± 1.4
Female	20.7 ± 1.5
Sex [*n* (% of female)]	163 (58.0)
Year of study [*n* (%)]	
II	222 (79.0)
III	59 (21.0)
Faculty [*n* (%)]	
Medicine	268 (95.4)
Dentistry	13 (4.6)
Family house [*n* (%)]	
village	61 (21.7)
a small town ^1^	62 (22.1)
a medium-sized city ^2^	58 (20.6)
big city ^3^	100 (35.6)
Type of place of residence [*n* (%)]	
student residence hall	47 (16.7)
rented room (in a shared apartment)	89 (31.7)
rented/owned apartment	94 (33.5)
family house	51 (18.1)
Surroundings of the place of residence [*n* (%)]	
noisy	112 (39.9)
peaceful	151 (53.7)
moderate traffic levels	13 (4.6)
other	5 (1.8)

^1^ <50,000 inhabitants; ^2^ 50,000–100,000 inhabitants; ^3^ >100,000 inhabitants.

**Table 2:** Statistically significant differences in anthropometric and body composition parameters of the studied group depending on sex.

Characteristics	Total [Mean ± SD]	Men [Mean ± SD]	Women [Mean ± SD]	Test Value ^1^
Height [cm]	172.2 ± 9.1	180.2 ± 6.7	166.4 ± 5.5	19.1 ^2^
Weight [kg]	65.2 ± 12.4	75.0 ± 10.1	58.1 ± 8.5	15.5 ^2^
Basal metabolic rate [kJ]	6639.7 ± 1248.1	7872.7 ± 872.0	5747.1 ± 484.3	26.1 ^2^
Fat percentage [%]	19.9 ± 6.5	15.4 ± 4.8	23.1 ± 5.6	−12.1 ^2^
Fat mass [kg]	13.0 ± 5.3	11.9 ± 4.9	13.8 ± 5.5	−3.6 ^2^
Fat-free mass [kg]	52.2 ± 10.7	63.1 ± 6.9	44.3 ± 3.9	28.9 ^2^
Total body water [kg]	37.7 ± 7.7	45.6 ± 4.7	32.0 ± 2.8	30.4 ^2^
Water percentage [%]	57.9 ± 5.0	61.2 ± 4.2	55.5 ± 4.0	11.6 ^2^
Predicted muscle mass [kg]	49.6 ± 10.2	60.0 ± 6.6	42.1 ± 3.7	28.9 ^2^
Muscle percentage [%]	76.1 ± 6.2	80.3 ± 4.6	73.0 ± 5.3	12.2 ^2^
Body mass index	21.8 ± 2.9	23.0 ± 2.4	21.0 ± 3.0	6.7 ^2^
ECW/ICW ^4^	0.677 ± 0.042	0.651 ± 0.039	0.696 ± 0.033	−10.4 ^2^
PA ^5^ [°]	6.0 ± 0.7	6.6 ± 0.5	5.5 ± 0.5	17.4 ^2^
Bone mass [kg]	2.6 ± 0.5	3.1 ± 0.3	2.3 ± 0.2	13.9 ^3^
Metabolic age [years]	17.0 ± 7.3	18.4 ± 8.0	16.0 ± 6.5	2.2 ^3^

^1^ at the level of statistical significance *p* < 0.001; ^2^ Student’s *t*-test; ^3^ Mann-Whitney U test; ^4^ Extracellular water/Intracellular water; ^5^ phase angle.

**Table 3:** Statistically significant (*p* < 0.05) differences in the lifestyle components of the studied group depending on sex.

Characteristics Consuming Frequency	Total [Mean ± SD]	Men [Mean ± SD]	Women [Mean ± SD]	U Test Value ^1^	*p* ^2^
Energy drinks [times/month]	1.4 ± 2.9	2.0 ± 3.3	0.9 ± 2.4	3.5	0.001
Cola [times/week]	1.0 ± 3.0	1.2 ± 2.5	0.9 ± 3.3	3.8	0.000
Beer [times/week]	1.1 ± 1.6	1.6 ± 2.0	0.7 ± 1.1	5.4	0.000
Wine [times/week]	0.5 ± 0.9	0.4 ± 0.8	0.6 ± 0.9	−3.2	0.001
Alcoholic drinks [times/week]	0.3 ± 0.6	0.3 ± 0.7	0.3 ± 0.5	−2.8	0.006
spirits-based alcohols [times/week]	0.5 ± 1.5	0.7 ± 1.5	0.4 ± 1.4	4.2	0.000
Water [l/day]	1.5 ± 0.7	1.7 ± 0.7	1.4 ± 0.7	3.6	0.000
Interval between meals [h]	4.1 ± 1.1	4.4 ± 1.1	3.9 ± 1.0	3.6	0.000
Vegetables/fruits [times/day]	1.7 ± 1.3	1.4 ± 1.1	2.0 ± 1.4	−3.9	0.000
Meat products [times/day]	1.2 ± 1.0	1.5 ± 0.9	1.0 ± 1.0	4.8	0.000
Fast food [times/month]	3.8 ± 6.2	5.1 ± 7.0	2.8 ± 5.3	4.1	0.000
Time					
Time for physical exercises [h/day]	58.7 ± 52.2	72.1 ± 65.3	49.0 ± 37.6	3.6	0.000
Watching TV [h/day]	0.4 ± 2.3	0.4 ± 3.4	0.4 ± 0.8	−2.2	0.030
Using a smartphone [h/day]	2.1 ± 1.6	1.7 ± 1.5	2.3 ± 1.7	−3.5	0.001
Using a computer [h/day]	1.8 ± 1.4	2.1 ± 1.5	1.7 ± 1.3	2.1	0.036

^1^ Mann-Whitney U test; ^2^ statistical significance.

**Table 4:** Significant correlations (*p* < 0.05) between PA and anthropometrics and body composition parameters.

Pearson’s Correlation (r)			
Body Composition Parameters	PA ^1^		
	Total	Men	Women
Height	0.461	n.s.	−0.200
Weight	0.565	n.s.	n.s.
Basal metabolic rate	0.681	0.212	0.187
Fat percentage	−0.432	n.s.	n.s.
Fat mass	−0.122	n.s.	n.s.
Fat-free mass	0.696	0.216	0.205
Total body water	0.711	0.263	0.207
Water percentage	0.431	n.s.	n.s.
Predicted muscle mass	0.696	0.216	0.205
Muscle percentage	0.436	n.s.	n.s.
Body mass index	0.443	0.303	0.255
ECW/ICW ^2^	−0.594	−0.475	−0.254
Bone mass	0.689 ^3^	0.197 ^3^	0.181 ^3^

^1^ phase angle; ^2^ Extracellular water/Intracellular water; ^3^ Spearman’s correlation; n.s.—statistically non-significant.

**Table 5:** Significant correlations (*p* < 0.05) between PA and components of lifestyle.

Spearman’s Correlation (r)			
Lifestyle Components	PA ^1^		
	Total	Men	Women
Energy drinks consumption	0.215	0.254	n.s.
Cola consumption	0.197	n.s.	n.s.
Beer consumption	0.284	n.s.	n.s.
Alcoholic drinks consumption	−0.135	n.s.	n.s.
spirits-based alcohols consumption	0.168	n.s.	n.s.
Water consumption	0.214	n.s.	n.s.
Interval between meals	0.215	n.s.	n.s.
Vegetables/fruit consumption	−0.144	n.s.	n.s.
Meat products consumption	0.254	n.s.	n.s.
Physical exercise frequency	0.164	n.s.	n.s.
Training time	0.227	n.s.	0.179
Time watching TV, using a smartphone and computer	−0.149	n.s.	n.s.

^1^ phase angle; n.s.—statistically non-significant.

**Table 6:** Odds ratios (ORs) and 95% confidence intervals (CIs) for PA relative to the factors analyzed.

Factors	OR	95% CI	*p* ^1^
Weight	1.39	1.21–1.59	0.000
Meat product consumption	1.12	1.02–1.24	0.020
Height	0.80	0.71–0.89	0.000
Fat percentage	0.77	0.68–0.87	0.000

^1^ statistical significance.

## Data Availability

The data presented in this article are available from the corresponding author on reasonable request.
